# Global prevalence of *Mycobacterium massiliense* strains with recombinant *rpoB* genes (Rec-Mas) horizontally transferred from *Mycobacterium abscessus*: two major types, dominant circulating clone 7 and MLST ST46 sequence type

**DOI:** 10.1128/spectrum.01935-24

**Published:** 2024-10-21

**Authors:** Dong Hyun Kim, Hyejun Seo, Sangkwon Jung, Bum-Joon Kim

**Affiliations:** 1 Department of Microbiology and Immunology, College of Medicine, Seoul National University, Seoul, South Korea; 2 Department of Biomedical Sciences, College of Medicine, Seoul National University, Seoul, South Korea; 3 Cancer Research Institute, College of Medicine, Seoul National University, Seoul, South Korea; 4 Seoul National University Medical Research Center (SNUMRC), Seoul, South Korea; 5 BK21 FOUR Biomedical Science Project, Seoul National University College of Medicine, Seoul, South Korea; ICON plc, London, United Kingdom

**Keywords:** *Mycobacterium abscessus*, horizontal gene transfer (HGT), *rpoB*, dominant circulating clone 7 (DCC7), ST46

## Abstract

*Mycobacterium abscessus* is a group of emerging antimicrobial-resistant nontuberculous mycobacteria that causes severe lung disease in infected patients globally. Recently, molecular epidemiology studies have indicated that horizontal gene transfer (HGT) events in the *rpoB* gene are prevalent between *M. abscessus* subspecies. To determine the global prevalence of *M. abscessus* strains subjected to *rpoB* HGT, we performed phylogenetic inference using a 711-bp *rpoB* sequence extracted from 1,786 *M*. *abscessus* isolates for which the whole-genome sequence was publicly available. Our data showed that a total of 74 isolates (4.1%) from 1,786 strains are subject to *rpoB* HGT, which is more prevalent than strains with *hsp65* HGT (19 isolates from 1,786, 1.1%). Most of these (69 isolates) belong to two major groups of *Mycobacterium massiliense*, of which the *rpoB* gene is horizontally transferred from *M. abscessus* (Rec-mas), dominant circulating clone 7 (DCC7) (44 isolates) and ST46 type by multilocus sequence typing (25 isolates). The Rec-mas strains of the two groups have distinct geographical patient distributions, of which the former is mainly distributed in the United States, while the latter is prevalent in Asia. Our further genome-based analysis indicated that the ST46 type is a novel DCC candidate of *M. massiliense* that is responsible for dissemination between noncystic fibrosis patients in Asia. In conclusion, our global phylogenetic analysis revealed two major Rec-mas clones with distinct geographical distributions, namely, DCC7 and ST46. This study provides insights into the genetic clustering and person-to-person transmission of globally dominant and area-specific strains harboring the HGT *rpoB* gene.

**IMPORTANCE:**

Horizontal gene transfer (HGT) events play a pivotal role in the evolution of *Mycobacterium abscessus* into dominant circulating clones (DCCs), which is capable of causing patient-to-patient transmission. In particular, HGT of the rpoB gene between strains of different subspecies of *M. abscessus* could also compromise differentiation between strains of *M. abscessus*. Here, for the first time, using 1,786 *M. abscessus* genome sequences, we evaluated the global prevalence of *M. abscessus* strains subjected to *rpoB* HGT. We found a greater prevalence of *M. abscessus* subjected to *rpoB* HGT than to those subjected to *hsp65* HGT, which is mainly due to two Rec-mas clones, dominant circulating clone 7 and ST46, which are responsible for dissemination between non-CF patients in Asia. Our data highlight the importance of *rpoB* HGT in the evolution of *M. abscessus*, particularly *Mycobacterium massiliense*, into virulent DCC clones.

## INTRODUCTION

*Mycobacterium abscessus*, a group of rapidly growing nontuberculous mycobacteria (NTM), has become a global health concern, threatening both healthy people and those with compromised immune systems (1). It is composed of three distinct subspecies: *M. abscessus* sub. *abscessus*, *Mycobacterium* sub. *massiliense*, and *Mycobacterium* sub. *bolletii* (2, 3). *M. abscessus* is notoriously known for being resistant to many antibiotics and difficult to treat (4, 5). Since there are differences between subspecies in terms of clinical severity and antibiotic resistance, separation between subspecies is important (6, 7).

Unlike nearly all NTM strains that are present as living environmental saprophytes, *M. abscessus* has evolved to cause indirect human-to-human infection, eventually leading to the emergence and global spread of genetically clustered clones with high virulence potential, the so-called dominant circulating clone (DCC) (8). Since the first identification of DCCs in sub. *abscessus* and sub. *massiliense*, seven distinct DCC types (DCC1 to DCC7) have been categorized, based on highly genetically related isolates from 20 different patients across multiple continents (9, 10). These DCCs account for more than 70% of infections in cystic fibrosis (CF) patients. Of these, DCCs 1 and 2 from sub. *abscessus* and 3 from sub. *massiliense* are found in the majority of patients suffering from CF patients worldwide (11, 12). To date, genome-based molecular epidemiologic studies regarding the global distribution of DCCs have focused mainly on Western nations, in which CF patients are prevalent, rather than Asian nations, in which *M. abscessus* infections with CF symptoms have rarely been found (13). Recently, the incidence of *M. abscessus* infection has also increased in Asian countries, including South Korea (14). Therefore, genome-based characterization of genetically clustered clones prevalent in Asian nations has gained attention for proper treatment and prevention.

Horizontal gene transfer (HGT) is recognized as a crucial process for bacterial genetic diversification, enabling survival in adverse environments (15). HGT can contribute to the evolution of tuberculosis-causing obligate human pathogens from environmental saprophytic NTM. HGT has been reported to play a pivotal role in the radical evolution of *M. abscessus* with unique features capable of causing indirect human-to-human transmission (16). For example, HGT events in which *dpnM* affects global gene expression could contribute to the emergence and global transmission of DCC1, 2, and 3, possibly by affecting fitness and adaptation to hosts (17). In addition, HGTs can supply genes even from unrelated species to a bacterial genome, affecting traits such as virulence and antibiotic resistance (18). Furthermore, inter-subspecies HGT events involving the *rpoB* gene between sub. *abscessus* and sub. *massiliense*, a major target for *M. abscessus* identification, have been found with a notable frequency, which could compromise *rpoB*-based subspecies identification of *M. abscessus* (19). Indeed, recent multilocus sequence typing (MLST) and genome-based studies have indicated that *M. abscessus* subspecies differentiation based on *rpoB* typing leads to misidentification of approximately 10%, with up to 20% in strains of sub. *massiliense*, suggesting that *rpoB* typing for *M. abscessus* identification is limited at the subspecies level and that a greater proportion of *M. abscessus* strains subject to *rpoB* HGT may be identified (19).

This study utilizes genomic data from globally registered *M. abscessus* isolates to identify subspecies that present classification challenges due to HGT of the *rpoB* gene. The research focuses on the global distribution of these *rpoB* HGT strains. Additionally, HGT events were investigated between DCCs, with an emphasis on the transfer of the *rpoB* gene, and include a functional categorization of the genes within the recombinant sequences. The findings from this study will aid in preventing misclassification due to *rpoB* gene transfer and provide insights into the genetic characteristics of *rpoB* HGT strains.

## MATERIALS AND METHODS

### Bacterial sample collection

One hundred and six strains of sub. *massiliense* were collected from different patients at the Asan Medical Center (Seoul, Republic of Korea) between 2004 and 2011. Six strains were isolated and typed by HGT from sub. *massiliense* carrying the recombinant sub. *abscessus rpoB* gene used in previous studies (20). Two of these strains, 50375 and 56120, were able to be cultured and used for whole-genome sequencing. This work was approved by the institutional review board of the Asan Medical Center (2012–0170), and informed consent was waived.

### Genomic DNA extraction

Mycobacterial genomic DNA was obtained from colonies grown on 7H10 agar plates supplemented with 0.2% glycerol and 10% OADC for 2 to 4 weeks. For cell lysis, cells from five loopfuls were suspended in 200 µL of lysis buffer (15% sucrose, 0.05 M EDTA, and 0.05 M Tris-Cl, pH 8.0) and briefly vortexed. The lysis buffer was prefiltered and kept at room temperature. Lysozyme was added to a final concentration of 20 mg/mL, and the mixture was incubated overnight at 37°C with shaking at 160 rpm. The next day, 6.25 µL of proteinase K (20 mg/mL), 100 µL of 20% SDS, and 8 µL of RNaseA/T1 were added, followed by a 10-minute incubation at 65°C. For DNA purification, an equal volume of phenol-chloroform-isoamyl alcohol (25:24:1) was added, mixed thoroughly, and then centrifuged at 13,000 rpm for 10 minutes at 4°C. The supernatant was collected, mixed with 0.6 volumes of isopropanol, and stored at −20°C for 30 minutes. After centrifugation, the DNA pellet was washed with 70% ethanol, dried, and resuspended in 20 µL of TE buffer (10 mM Tris-Cl, 1 mM EDTA, pH 8.0) for further use.

### Whole-genome resequencing

Whole-genome resequencing and subsequent analysis were outsourced to Macrogen Inc. (Seoul, South Korea). The sequencing libraries were prepared according to the manufacturer’s instructions for the TruSeq Nano DNA High Throughput Library Prep Kit (Illumina) (21). Briefly, 100 ng of genomic DNA was sheared using adaptive focused acoustic technology (Covaris), and the fragmented DNA was end-repaired to create 5′-phosphorylated, blunt-ended dsDNA molecules. Following end repair, the size of the DNA was selected via a bead-based method. These DNA fragments were subjected to the addition of a single “A” base and ligation of the TruSeq DNA UD Indexing adapters. The products are then purified and enriched via PCR to create the final DNA library. The libraries were quantified using quantitative polymerase chain reaction (qPCR) according to the qPCR Quantification Protocol Guide (KAPA Library Quantification kits for Illumina Sequencing platforms) and qualified using the Agilent Technologies 4200 TapeStation D1000 screentape (Agilent Technologies). Then, paired-end (2 × 150 bp) sequencing was performed by Macrogen using NovaSeq X (Illumina). The reads were sequenced using the *M. abscessus* ATCC19977 type strain (NC_010397.1) as a reference genome. The sequence data have been deposited in the Sequence Read Archive (SRA) under BioProject number PRJNA1133902.

### Genome data set construction

A previously constructed set of 1,786 public assemblies of *M. abscessus* was downloaded from the NCBI assembly database (22). Additional *M. abscessus* strains of Asian origin were obtained from BioProjects PRJDB10333, PRJDB10566, and PRJNA734660 (13, 23, 24). The raw reads were downloaded from the SRA as FastQ files and processed on a Linux Ubuntu 22.04.3 LTS server. *De novo* assembly was performed using Shovill v1.1.0 under standard settings, and the data were prepared for subsequent analyses (https://github.com/tseemann/shovill). Read trimming and correction were carried out using Trimmomatic v0.39 and Lighter v1.1.2, respectively (25, 26). Within the Shovill pipeline, both SKESA v2.4.0 and SPAdes v3.15.0 served as assembly tools (27, 28). Sequence typing for each strain was then performed using the seven-loci *abscessus* scheme (*argH*, *cya*, *gnd*, *murC*, *pta*, *purH*, and *rpoB*) in pubMLST (29).

### Phylogenetic analysis

BLAST was used to extract the *rpoB* and *hsp65* genes from 1,786 *M*. *abscessus* isolates. All partial sequences of the *rpoB* (711 bp) and *hsp65* (603 bp) genes were aligned using MAFFT software (30). Subsequently, a phylogenetic tree was constructed utilizing the maximum likelihood method implemented in FastTree 2.1.11 (31). The phylogeny was constructed using the Jukes-Cantor model, applying 20 rate categories per site. To construct a phylogenetic tree for the *M. abscessus* using whole-genome sequences (WGS), PhaME software was used (32). The FastTree method implemented in the software was employed to generate whole genome-based phylogeny. The analysis included 20 strains from each DCC and 25 strains of sequence type ST46. *M. abscessus* ATCC19977 (NC_010397.1) was used as a reference sequence.

### Pairwise SNP distance

Genomic sequences were aligned against the annotated reference genome *M. abscessus* ATCC19977 (NC_010397.1) using Parsnp v1.2 (33). Core genome alignment was performed for 301 strains of DCC3, 36 strains of DCC6, 41 strains of DCC7, and 56 strains of ST46. The software aligned multiple genomes to identify core genome single nucleotide polymorhpisms (cgSNPs) and returned the results in the form of a FASTA file. The resulting SNP alignment was then used for pairwise SNP distance calculations with the snp-dists (https://github.com/tseemann/snp-dists).

### Clustering and transmission network analysis

Clustering and transmission network analyses of *M. abscessus* strains were performed using GraphSNP (v1.0) (34). For both analyses, metadata such as collection date and geographic location data were organized using NCBI BioSample numbers. Both metadata and SNP distance data were used as inputs. For clustering analysis, a 38-SNP cutoff was applied. This cut-off value was established considering the collection period (2004–2017) and the geographic regions (Europe, America, and Asia) from which ST46 samples were obtained. The CATHAI method with a COSE layout was used for clustering visualization (35). Transmission network analysis employed the SeqTrack algorithm to construct a parsimonious transmission tree based on SNP distances and sample collection dates, with a predefined SNP threshold filtering out unlikely transmission links (36).

### Antimicrobial resistance mutation and pangenome analysis

To identify antimicrobial resistance variants, the positions of rRNA genes in the genome of each strain were predicted using Barrnap v0.9 (https://github.com/tseemann/barrnap). All rRNA genes were annotated in DCC7 and ST46. Specific mutations, A2269C and A2270G, were detected in the *rrl* sequences of all annotated strains. The genomic sequences of all *M. abscessus* strains were annotated using Prokka v1.14.6 with default settings, which produced GFF3 files (37). These annotated genomes were input into Roary v3.13.0, which clustered genes into orthologous groups to construct the pangenome (38).

### Recombination detection

For the recombination detection analysis, five strains each from DCC and sequence type ST46 were randomly selected, resulting in a total of 40 strains. The core genomes of these strains were aligned, covering 81% of the reference *genome M. abscessus* ATCC 19977 (5,067,172 bp). This alignment was analyzed using the Recombination Detection Program v5 under default settings (39). For different subspecies, only recombination events detected by more than six algorithms (RDP, GENECONV, Bootscan, MaxChi, Chimera, and SiScan) and deemed statistically significant were considered.

### Characterization of the HGT region

The recombination detection results revealed the genes transferred from sub. *abscessus* to sub. *massiliense*. These recombinant regions were translated into protein sequences and submitted to the KEGG BlastKOALA server for functional annotation (40). The sequences were classified based on their functional annotations, with KO identifiers assigned to orthologous genes via BlastKOALA. Pathway reconstruction was conducted using the online KEGG Mapper-Reconstruction tool with the obtained KO numbers. The recombinant regions were identified and extracted according to their positions in the *M. abscessus* ATCC19977 reference genome. In DCC3, 392 coding domains were identified, while 275 coding domains were found in ST46.

### Statistical analysis

Statistical analysis and image visualization were conducted using GraphPad Prism 10. A *P* value less than 0.05 was considered to indicate a statistically significant difference.

## RESULTS

### Global prevalence of *M. massiliense* strains subjected to HGT of the *rpoB* gene (Rec-mas strains)

To evaluate the global prevalence of *M. abscessus* subjected to HGT of the *rpoB* gene, a total of 1,786 publicly available genome sequences of *M. abscessus* isolates were used to construct a 711-bp partial *rpoB*-based phylogeny. This identification was compared with a 603-bp *hsp65*-based phylogeny. The *hsp65*-based phylogeny revealed that out of the 1,108 sub. *abscessus*, 28 misidentified sub. *abscessus* strains (16 as sub. *massiliense*, 12 as sub. *bolletii*), with 97.47% agreement (Table 1). Among sub. *massiliense* strains, three were misidentified, showing 99.46% agreement. The overall agreement for hsp65-based differentiation was 98.26%. According to *rpoB*-based typing, 5 sub. *abscessus* strains were misidentified (99.55% agreement), while 74 sub. *massiliense* strains were misidentified (86.67% agreement). The overall agreement for *rpoB*-based differentiation was 95.58%. HGT of the *rpoB* gene was predominantly observed from sub. *abscessus* to sub. *massiliense*, with no strains of rpoB gene transfer detected in sub. *bolletii*.

**TABLE 1:** Comparison between identification methods based on the whole-genome SNP and *rpoB* and *hsp65* genes for subspecies differentiation between the *M. abscessus* and their HGT frequency^*a*^

Typing gene	Subspecies	cgSNP based	Identical strain	Unidentical strain	Recombination between		Agreement (%)
					ABS and MAS	BOL and ABS	
*hsp65*	*M. abscessus*	1108	1080	28	16	12	97.47%
	*M. massiliense*	555	552	3	3		99.46%
	*M. bolletii*	123	123	0			100%
	Total	1786	1755	31	19	12	98.26%
*rpoB*	*M. abscessus*	1108	1103	5	5		99.55%
	*M. massiliense*	555	481	74	74		86.67%
	*M. bolletii*	123	123	0			100%
	Total	1786	1707	79	79		95.58%

^*a*^ ABS, MAS, and BOL refer to *M. abscessus*, *M. massiliense*, and *M. bolletii*, respectively.

**Fig 1:**
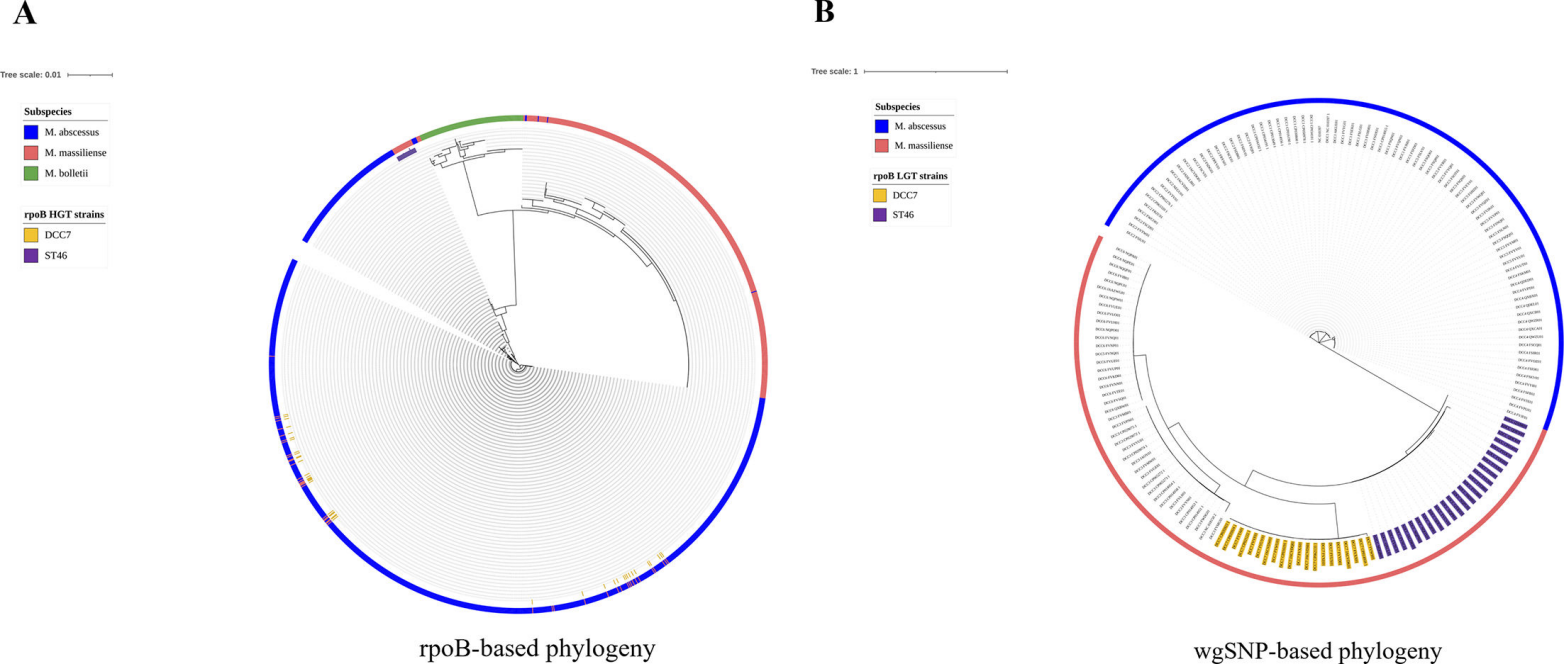
Phylogenetic location of two types of Rec-mas clones on the whole genome-based phylogenetic tree and the partial *rpoB* phylogenetic tree inferred by the FastTree method. Sub. *abscessus*, *massiliense*, and *bolletii* are annotated in blue, red, and green, respectively. The 41 Rec-mas strains written in yellow background tips belong to DCC7, and the 25 Rec-mas clones written in purple background tips are novel non-DCC strains. The scale bar represents the number of nucleotide substitutions per site. (**A**) A phylogenetic tree using the 711-bp partial *rpoB* gene extracted from the genome of the *M. abscessus* 1786 strain. (**B**) A phylogenetic tree using the whole-genome sequences of 20 strains randomly selected from each DCC (1 ~ 7) and 25 non-DCC Rec-mas strains.

Of the 74 sub. *massiliense* species discovered in the sub. *abscessus* branch (Rec-mas strains), the 66 strains belonged to two major groups (Fig. 1A). One cluster included a total of 41 strains with a 711-bp *rpoB* sequence identical to the sub. *abscessus* type strain ATCC19977. This cluster proved to belong to DCC7 of sub. *massiliense* using whole genome-based phylogenetic analysis (Fig. 1B). The other cluster included 25 strains with chimeric *rpoB* sequences identical to those of previously reported Rec-mas strains from South Korea (20), some of which were horizontally transferred from sub. *abscessus*. Further WGS-based cgSNP analysis indicated that all 25 strains harboring the chimeric *rpoB* gene formed a phylogenetically clustered branch belonging to the non-DCC type of sub. *massiliense*, indicating that some of the *rpoB* sequences of these strains were horizontally transferred from sub. *abscessus*.

When the MLST scheme was applied to the strains belonging to the two clusters, most strains (36/41 strains, 87.8%) belonging to DCC7 were of the ST42 type, and all 25 strains of non-DCC strains were of the ST46 type (Table 2). Collectively, our data indicated that there are two types of Rec-mas strains, DCC7 (41 strains/555, 7.4%) and ST46 (25 strains/555, 4.5%), among the global sub. *massiliense*.

**TABLE 2:** MLST sequence types of two Rec-mas types

Subspecies	DCC	Accession no.	No.	Sequence type	argH	cya	gnd	murC	pta	purH	rpoB
*M. massiliense*	DCC7	NZ_CP021122.1	36	42	30	31	19	28	16	15	1
		NZ_CP016191.1	2	103	1	31	19	28	16	15	1
		FVWS01	2	112	30	31	19	28	28	15	1
	Non-DCC	NZ_CP014952.1	25	46	30	7	17	30	21	31	10

### Geographic distribution of two Rec-mas types, DCC7 and ST46

The geographical distributions of the 41 strains of DCC7 and 25 strains of ST46 identified were analyzed to determine their global prevalence (Table 3). Notably, DCC7 is found in North America (10.1%, 33 strains/326) and Europe (0.8%, 8 strains/950), with no isolates in Asia or Oceania, indicating that DCC7 infection is most prevalent in North America. The 25 strains of ST46 were the most prevalent in Asia (4.0%, 12 strains/297). This finding suggests that there is a distinct disparity between the global geographical distributions of DCC7 and ST46.

**TABLE 3:** Global geographical distribution of two Rec-mas types

Project accession	Continent						Total	Related study
	Asia	North America	South America	Europe	Oceania	Unknown		
Curated BioProjects	297 (16.6%)	326 (18.3%)	8 (0.4%)	950 (53.2%)	195 (10.9%)	10 (0.6%)	1786 (100%)	Diricks et al., (22)
DCC7	0	33 (10.1%)	0	8 (0.8%)	0	0	41 (2.3%)	
ST46	12 (4.0%)	2 (0.6%)	1 (12.5%)	9 (0.9%)	1 (0.5%)	0	25 (1.4%)	

To verify the epidemiological features found in this study, Rec-mas strains from three additional Asian bioprocesses were further analyzed (Table 4) (13, 23, 24). Eleven additional ST46 clones were found in 263 patients from Japan (4.2%), 4 in 105 patients from Taiwan (3.8%), and 14 in 210 patients from Singapore (6.7%). No strains belonging to DCC7 were found in an additional 578 Asian patients. The prevalence of ST46 and DCC7 among the 875 Asian strains was 4.7% (41 strains/875) and 0% (0 strain/875), respectively. Collectively, our data indicate that the two Rec-mas types, DCC7 and ST46, show distinct geographical distributions, with the former prevalent in North America and the latter prevalent in Asia, suggesting that there are different global transmission networks between the two Rec-mas strains, DCC7 and ST46.

**TABLE 4:** Geographical distribution of two Rec-mas types between Asian nations in three additional Asian data sets

Project accession	Asia						Total	Related study
	China	Japan	Taiwan	Singapore	Malaysia	Etc.		
Curated BioProjects	279				11	7	297	Diricks et al. (22)
PRJDB10333		148					148	Yoshida et al. (24)
PRJDB10566		115	105				220	Yoshida et al. (13)
PRJNA734660				210			210	Chew et al. (23)
DCC7	0	0	0	0	0	0	0	
ST46	12 (4.3%)	11 (4.2%)	4 (3.8%)	14 (6.7%)	0	0	41 (4.7%)	
Total	279	263	105	210	11	7	875	

### ST46 transmission networks through non-CF patients in Asia

**Fig 2:**
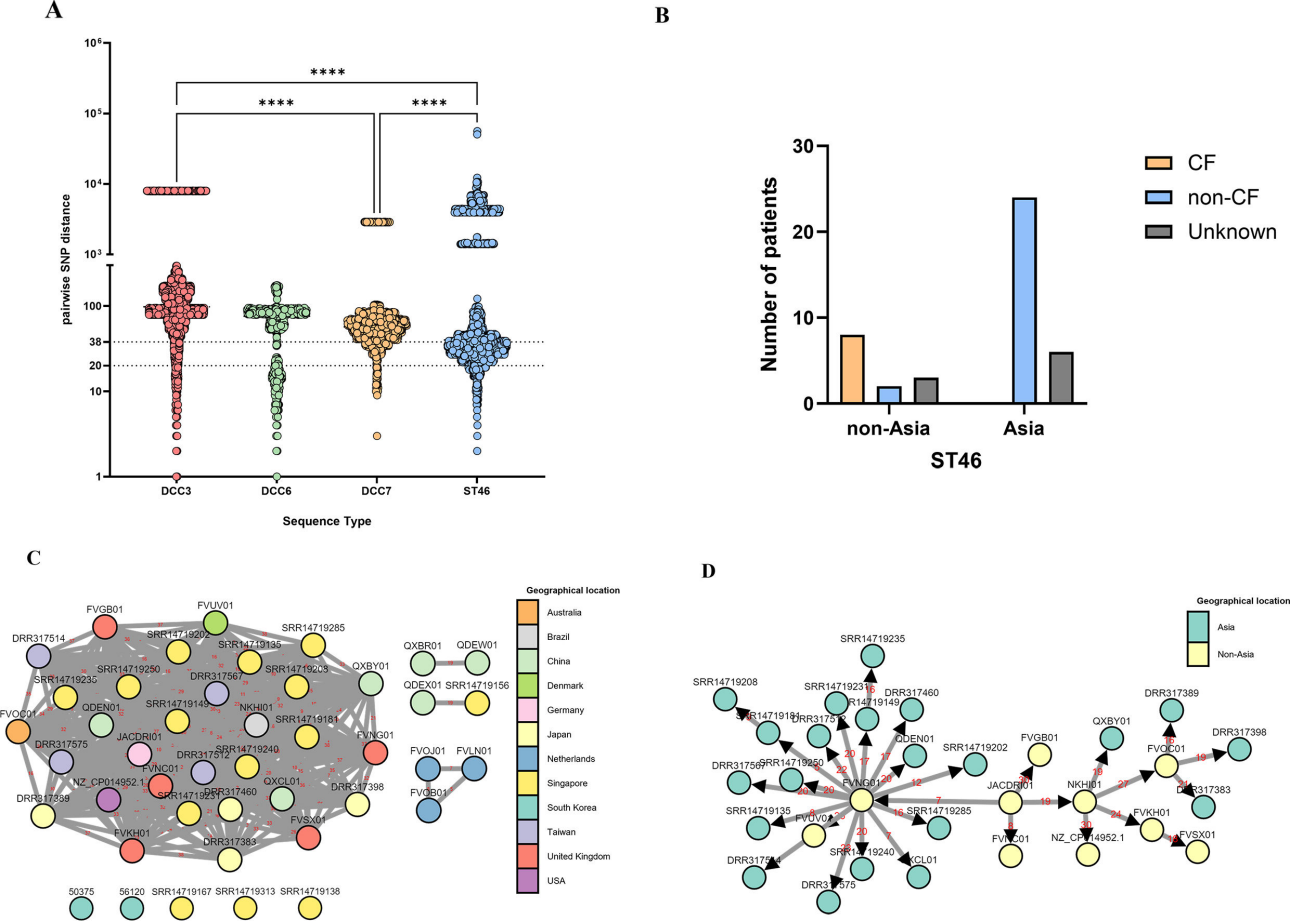
(**A**) Violin plot comparing pairwise SNP distances within the core genomes of the DCC3 (red), DCC6 (green), DCC7 (orange), and ST46 (blue) strains. The plot highlights the distribution and median SNP distances for each group. The horizontal dashed lines at 20 SNPs and 38 SNPs indicate genetic distances suggesting “probable” and “possible” transmission, respectively. (**B**) Bar graph showing the number of CF and non-CF patients with ST46 strains in Asian and non-Asian regions. CF patients are shown in orange, non-CF patients are shown in blue, and unknown patients are shown in gray. (**C**) Network graph showing the SNP clustering analysis of ST46 strains. Each node represents an individual strain, color coded by geographic region, with edges denoting SNP distances between strains. This analysis highlights the genetic relatedness and clustering of ST46 strains across different regions. (**D**) Transmission network diagram illustrating the potential transmission pathways of the ST46 strains. Nodes represent individual strains, with green nodes indicating strains of Asian origin and yellow nodes indicating strains of non-Asian origin. Arrows indicate the direction of possible transmission events, emphasizing the origins and spread of ST46 strains across various regions. Significant differences between groups are denoted by asterisks (****P < 0.0001).

To determine the genetic relatedness between the ST46 strains, we compared pairwise SNP distances between the ST46 strains and the other DCCs of sub. *massiliense*. A total of 56 ST46 strains (25 from global 1,786 *M*. *abscessus* strains, 29 strains from three additional BioProjects of Asian patients, and 2 strains from Korean patients for which WGS was conducted in this study), 301 strains of DCC3, 36 strains of DCC6, and 41 strains of DCC7 were used for this comparison. The pairwise SNP distances were calculated within the core genome of each DCC and ST46 (Fig. 2A). Our data indicated that compared with other DCCs, ST46 had the lowest median SNP value of 40 (range, 0–56,120), with a median of 98 SNPs in DCC3 (0–8,019), 76 SNPs in DCC6 (0–607), and 57 SNPs in DCC7 (0–2,896), suggesting close genetic relationships between ST46 strains.

Next, we investigated the relationships between cystic fibrosis and the geographical distribution of the ST46 Rec-mas strains. Our data indicated that there were no ST46 isolates from CF patients in Asia, whereas in non-Asian patients, 8 out of 13 strains were isolated from CF patients (Fig. 2B), suggesting the dissemination of ST46 between non-CF patients in Asia. To determine the genetic relatedness and transmission network between geographically divided individuals (Asian patients vs. non-Asian patients) with and without CF, clustering analysis and transmission network analysis were conducted using 43 ST46 strains available for collection date and geographic location (Table S1). When a cutoff value of 38 SNPs, indicating “possible” transmission, was used (8), a total of four clusters were produced (Fig. 2C). The largest cluster (Cluster I) included 31 strains, of which 21 (67.7%) were from Asia and 10 (32.3%) were from other regions (median of 29 SNPs; range, 5–48). We found no discrete transmission chain between CF patients and non-CF patients in Cluster I. However, all three CF patients from the Netherlands were clustered with a distinct transmission network chain, suggesting dissemination between CF patients in the Netherlands.

Transmission network analyses between Cluster I strains responsible for major dissemination in Southeast Asian countries, including Singapore and Taiwan (17/21, 81.0%), revealed that they originated from a strain from the UK in 2012 (WGS project no. FVNG01), which may have been transmitted from a strain discovered in Germany in 2000 (WGS project no. JACDRI) (Fig. 2D). Global transmission network analysis indicated that ST46 strains may have initially been transmitted from non-Asian areas, including the UK, Germany, or Brazil, before spreading to Asia.

### Mutation analysis of GPL biosynthesis-related and antimicrobial resistance genes in two Rec-mas types

To evaluate the genetic mutations associated with clinical outcomes in DCC7 and ST46 patients, a comparative genomic analysis was performed. Specifically, we focused on the genotyping of *erm* (41) deletions and mutations in the *rrl* gene, which encodes the 23S rRNA gene (Table 5). Among the 41 strains in the DCC7 group, 6 (14.6%) exhibited the A2269C mutation in the *rrl* gene. One strain (2.4%) was found to have the A2270G mutation. For the ST46 group, 3 out of 55 strains (5.5%) exhibited the A2269C mutation. There were no occurrences of the A2270G mutation in any of the ST46 strains. The *erm* (41) gene was deleted in both types of Rec-mas.

**TABLE 5:** Frequency of *rrl* resistance variants and *erm* (41) gene deletions in two Rec-mas types

Sequence type	*rrl* resistance variant		*erm* (41) gene presence
	A2269G (A2058N)	A2270G (A2059N)	
DCC7	6/41	1/41	0/41
ST46	3/56	0/56	0/56

Pangenome analysis revealed 263 strains from DCC3, 36 from DCC6, 41 from DCC7, and 53 from ST46. Using genes related to glycopeptidolipid (GPL) biosynthesis from *M. abscessus* ATCC19977 as a reference (41), the percentage of strains with deletions of GPL-related genes among the total strains of each DCC and ST was calculated. Mutations in GPL biosynthesis-related genes in the two Rec-mas types were analyzed (Table 6). Annotation was performed for each DCC and ST strain, and the proportions of strains lacking GPL-related genes in the Rec-mas and other *massiliense* DCCs were calculated. Multiple regions of GPL-related genes, especially the *mps1* gene (14.1%), were deleted in DCC3. DCC6 had 10 strains out of 36 (27.8%) with deletions in the *fadD23* gene (MAB_0935c). DCC7 had five strains with a deletion in the *mps1* gene (MAB_4099c). Only two ST46 strains had a GPL-related gene deletion (MAB_4098c), consistent with previous findings showing that all 6 Rec-mas strains from Korean patients have smooth colony morphotypes (20). Our data suggest that intact GPL-related genes found in ST46 strains (96.2%) could contribute to their survival in hostile environments, resulting in their dissemination between non-CF patients.

**TABLE 6:** Frequency of deleted GPL biosynthesis-related genes between DCCs and ST46 of sub. *Massiliense*

Gene	locus_tag	Name	DCC3	DCC6	DCC7	ST46
*gap-like*	MAB_0934	GAP family protein CDS	100.0%	100.0%	100.0%	100.0%
*fadD23*	MAB_0935c	AMP-binding protein CDS	100.0%	72.2%	100.0%	100.0%
*PE*	MAB_0936c	pe CDS	100.0%	94.4%	100.0%	100.0%
*mmpL10*	MAB_0937c	RND family transporter CDS	98.1%	100.0%	100.0%	100.0%
*papA3*	MAB_0938c	Condensation domain-containing protein CDS	100.0%	100.0%	100.0%	100.0%
*pks*	MAB_0939	Type I polyketide synthase CDS	98.9%	100.0%	97.6%	100.0%
*gap-like*	MAB_4097c	GAP family protein CDS	98.9%	100.0%	100.0%	100.0%
*mps2*	MAB_4098c	Nonribosomal peptide synthetase CDS	100.0%	100.0%	100.0%	96.2%
*mps1*	MAB_4099c	Nonribosomal peptide synthetase CDS	85.9%	100.0%	87.8%	100.0%
*mbtH*	MAB_4100c	MbtH family protein CDS	99.6%	100.0%	100.0%	100.0%
*fmt*	MAB_4103c	Class I SAM-dependent methyltransferase CDS	87.8%	100.0%	100.0%	100.0%
*gtf2*	MAB_4104	Glycosyltransferase CDS	100.0%	100.0%	100.0%	100.0%
*rmt3*	MAB_4105c	TylF/MycF/NovP-related O-methyltransferase CDS	100.0%	100.0%	100.0%	100.0%
*atf1*	MAB_4106c	Acyltransferase CDS	98.1%	100.0%	100.0%	100.0%
*gtf1*	MAB_4107c	Glycosyltransferase CDS	100.0%	100.0%	100.0%	100.0%
*rmt4*	MAB_4108c	TylF/MycF family methyltransferase CDS	100.0%	100.0%	100.0%	100.0%
*rmt2*	MAB_4109c	Class I SAM-dependent methyltransferase CDS	100.0%	97.2%	100.0%	100.0%
*atf2*	MAB_4110c	Acyltransferase CDS	100.0%	100.0%	100.0%	100.0%
*rmlB*	MAB_4111c	NAD-dependent epimerase/dehydratase family protein CDS	100.0%	100.0%	100.0%	100.0%
*gtf3*	MAB_4112c	Glycosyltransferase CDS	100.0%	100.0%	100.0%	100.0%
*rmlA*	MAB_4113	rfbA CDS	100.0%	100.0%	100.0%	100.0%
Rv1174	MAB_4114	Hemophore-related protein CDS	100.0%	100.0%	100.0%	100.0%
*mmpL4b*	MAB_4115c	RND family transporter CDS	100.0%	100.0%	100.0%	100.0%
*mmpL4a*	MAB_4116c	RND family transporter CDS	100.0%	100.0%	100.0%	100.0%
*mmpS4*	MAB_4117c	MmpS family transport accessory protein CDS	100.0%	100.0%	100.0%	100.0%
*fadE5*	MAB_4437	Acyl-CoA dehydrogenase CDS	100.0%	100.0%	100.0%	100.0%
*sap*	MAB_4454c	YciI family protein CDS	100.0%	100.0%	100.0%	100.0%
*ecf*	MAB_4459c	RNA polymerase sigma factor CDS	100.0%	100.0%	100.0%	100.0%
Rv0926	MAB_4633	Diacylglycerol kinase CDS	100.0%	100.0%	100.0%	100.0%
GPL-related gene deleted strain/total strain			44/263 (16.7%)	13/36 (36.1%)	5/41 (12.2%)	2/53 (3.8%)

### Molecular characterization of the interspecies HGT regions of the two Rec-mas types

**Fig 3:**
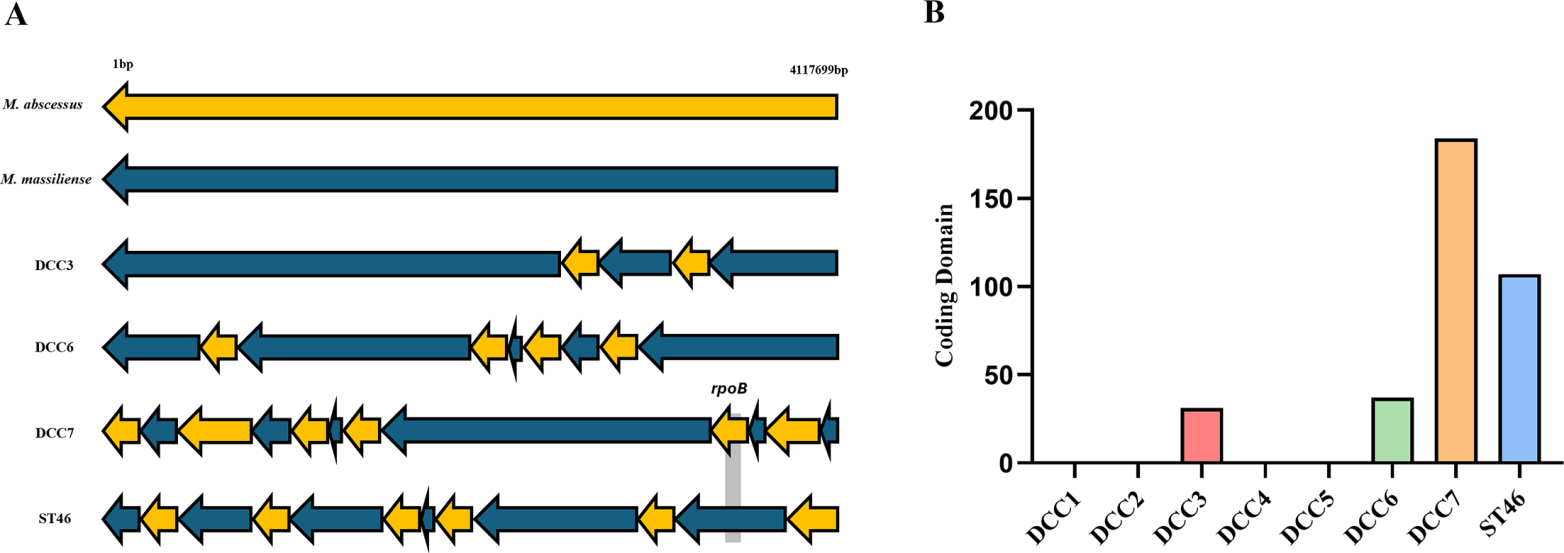
(**A**) Diagram showing the interspecies recombination occurrence and location of the rpoB gene. The horizontal arrows represent the genomes of sub. *massiliense*, DCC3, DCC6, DCC7, and ST46. Yellow arrows indicate regions transferred from sub. *abscessus* to sub. *massiliense*. The *rpoB* gene in DCC7 is highlighted in gray. (**B**) Bar graph depicting the number of recombinant coding domains identified in each DCC. DCC3 is shown in red, DCC6 in green, DCC7 in blue, and ST46 in orange. The y-axis represents the number of coding domains, emphasizing the greater frequency of recombination events in DCC7 and ST46.

Since HGT events between *M. abscessus* subspecies could have a great effect on their virulence and drug resistance (18), we investigated the molecular characteristics of inter-subspecies HGT events that occurred in DCCs and ST46. Interspecies recombination occurred only in sub. *massiliense* but not in sub. *abscessus*. In sub. *massiliense*, interspecies HGT events occurred two times in DCC3, four times in DCC6, and six times in DCC7 and ST46, indicating a greater frequency of inter-subspecies HGT events in the two types of Rec-mas (Fig. 3A). Specifically, 31 coding domains from sub. *abscessus* were transferred into the HGT region of DCC3, 37 into the HGT region of DCC6, 184 into the HGT region of DCC7, and 107 into the HGT region of ST46 (Fig. 3B). Notably, the HGT of the *rpoB* gene in DCC7 was located in the fifth HGT region, covering 105,659 bp (Table S3). The *rpoB* gene of ST46 was partially transferred, as previously reported (20)

Through core genome alignment, the HGT regions of DCC7 and ST46 transferred from sub. *abscessus* were functionally categorized (Fig. S1). When the DCC7 recombinant gene was annotated against the KEGG pathway, 41.8% (164 out of 392) of the functional categories could be retrieved. The essential genes related to genetic information processing, such as *rpoB* and *rpoC*, account for the largest proportion of horizontally transferred genes (22%), which can affect the global expression of bacterial genes, possibly leading to the emergence of virulent clones. Among the ST46 recombinant genes, 31.6% (87 out of 275) were retrieved. Only 5% of genes is subject to the genetic information processing category. Notably, four copies of the mmpl4 coding domain in the HGT region of ST46 were transferred from sub. *abscessus* DCC. It has been reported to play a pivotal role in mycobacterial pathogenesis and GPL biosynthesis as a *Mycobacterium* membrane protein (42), contributing to the capacity for human transmission via enhanced survival in hostile environments.

## DISCUSSION

Recent global genome studies have indicated that *M. abscessus*, a drug-resistant NTM, has evolved into genetically homogenous virulent clones, DCCs, via HGT gene acquisition. Although in general, HGT events involving essential genes such as the *rpoB* gene rarely occur, once HGT occurs, HGT can have a great effect on global gene transcription and expression in recipient cells, leading to a fundamental change in the course of their evolution (17). Additionally, this could compromise the potential of *rpoB* sequence-based methods for bacterial diagnosis or identification. Our previous genome-based phylogenetic study demonstrated HGT acquisition of the *rpoB* gene of *Mycobacterium intracellulare* sub. *yongonense* from *Mycobacterium parascrofulaceum*, which is a distantly related scotochromogen, suggesting its pivotal role in mycobacterial evolution (43, 44). Moreover, we also reported that *rpoB* HGT events occur in some strains of sub. *massiliense* (Rec-mas), which could compromise *rpoB*-based diagnosis for *M. abscessus* subspecies identification (19). Distinguishing between sub. *abscessus* and sub. *massiliense* is crucial for clinical practice due to differences in antibiotic resistance genes. Sub. *abscessus* carries the *erm* (41) gene, which provides resistance to macrolide antibiotics, while sub. *massiliense* lacks this gene due to a deletion. Rapid and accurate identification is essential for effective treatment, as misidentifying sub. *massiliense* as sub. *abscessus* could delay appropriate therapy and impact patient outcomes.

In this study, we hypothesize that the effect of *rpoB* HGT on global gene expression could lead to the emergence of genetically clustered virulent clones. Therefore, we investigated the global prevalence of Rec-mas strains and elucidated their epidemiologic and genomic characteristics. First, we found that *rpoB* HGT is more frequently found in global *M. abscessus* strains than the *hsp65* gene [4.42% (79 isolates/1786) vs. 1.74% (31 isolates/1786)] and it is mainly found in sub. *massiliense* strains and Rec-mas strains [93.67%, (74 isolates/79)], rather than the other two subspecies, supporting our previous finding indicating a greater possibility of misidentification in the application of the *rpoB* single gene for MAB identification (20) compared with the *hsp65* gene (Table 1). Two epidemiologically and genetically distinct Rec-mas clones were identified: DCC7, which is found exclusively in Western countries, and another Rec-mas clone that is prevalent in Asian countries (Fig. 1 and 2). According to MLST analysis, DCC7 predominantly corresponds to ST42, while the Rec-mas clone prevalent in Asia is classified as ST46. To conserve computational resources and time, the prevalence of both Rec-mas clones was confirmed using MLST with additional Asian BioProjects rather than analysis based on WGS (Tables 2 and 4). Notably, a total of 56 strains of the ST46 type had 711 bp identical chimeric *rpoB* sequences and identical MLST patterns, as previously shown for six Rec-mas isolates of the smooth colony morphotype from Korean patients (Table 4) (20). In this study, phylogenetic analysis of two Korean Rec-mas strains also revealed that they belonged to the ST46 type (Table 2).

Our inter-subspecies recombination analysis revealed recombination events only in the DCCs of sub. *massiliense* (DCC3, 6, 7) but not in sub. *abscessus* (DCC1, 2, 4, and 5), consistent with previous findings (Fig. 3A) (19). The cgSNP-based analysis revealed that the two Rec-mas types, DCC7 (184 coding domains) and ST46 (107 coding domains), possess a greater number of recombined coding domains compared with DCC3 (31 domains) and DCC6 (37 domains) (Fig. 3B). Among the domains transferred by HGT events in the DCC7 type, several housekeeping genes, including *rpoB*, *rpoC*, and *rplV*, are involved mainly in global transcription from sub. *abscessus*, which could contribute to the emergence of a virulent ancestor of DCC7 from an environmental ancestor (Fig. S1). Of the domains transferred by HGT events in the ST46 type, four *mmpl4* genes were transferred from sub. *abscessus*, which have been reported to play a pivotal role in virulence and drug efflux in *M. tuberculosis* (45). It can contribute to the virulence of *M. abscessus* via the transfer of GPL to the outer mycobacterial surface (42, 46, 47). Consistent with this finding, all six Rec-mas isolates from Korean patients were smooth colony types, suggesting that they had intact GPLs (20). Moreover, our genomic investigation revealed no mutations in the GPL loci in the majority of the ST46 type (96.2%) (Table 6), suggesting that these traits contribute to the increased virulence of the ST46 type via fomite-mediated indirect human-to-human transmission. Therefore, it is tempting to speculate that the acquisition of genetic diversity by inter-subspecies HGT events could contribute to increased infectivity or host adaptation of two Rec-mas types, DCC7 and ST46, resulting in the emergence of genetically homogeneous virulent clones via radical evolution (17).

Pairwise SNP distance analysis indicated that 56 strains belonging to the ST46 Rec-mas type were more genetically clustered between isolates (median SNP level: 40) than other *massiliense* DCCs, including DCC7, suggesting that it may be a novel DCC type of sub. *massiliense* (Fig. 2A). Notably, our data also showed that strains of this type first originated from Germany and then spread to Brazil or the United Kingdom, after which they became widespread in Asian nations (Fig. 2C and D). Given that CF patients are rare in Asian nations, the ST46 Rec-mas type may be a distinct trait capable of indirect human-to-human transmission between non-CF Asian patients (Fig. 2B). This issue should be explored in future studies.

In conclusion, our global phylogenetic analysis of *M. abscessus* strains indicated that there are two major sub. *massiliense* clones subjected to *rpoB* HGT, Rec-mas-type clones with distinct patient geographical distributions, a Western-specific DCC7 and an Asian-prevalent ST46, which could compromise identification by single *rpoB*-based diagnosis for *M. abscessus*. Our further SNP and interspecies HGT analyses indicated that the ST46 Rec-mas type must be classified as a novel DCC of the sub. *massiliense*, which is responsible for dissemination between non-CF Asian patients. This study provides insights into the genetic clustering and person-to-person transmission of globally dominant and area-specific *M. abscessus* strains harboring the HGT *rpoB* gene.

## Data Availability

The WGS data produced in this study can be accessed in the NCBI SRA under the BioProject accession number PRJNA1133902.

## SUPPLEMENTAL MATERIAL

Fig. S1 spectrum.01935-24-s0001.pdf Functional category of recombinant genes.

Supplemental tables spectrum.01935-24-s0002.xlsx Tables S1 to S3.

ASM does not own the copyrights to Supplemental Material that may be linked to, or accessed through, an article. The authors have granted ASM a non-exclusive, world-wide license to publish the Supplemental Material files. Please contact the corresponding author directly for reuse.
