# Supplementary material for: Global prevalence of Mycobacterium massiliense strains with recombinant rpoB genes (Rec-Mas) horizontally transferred from Mycobacterium abscessus: two major types, dominant circulating clone 7 and MLST ST46 sequence type
Source: Microbiol Spectr. 2024 Oct 21;12(12):e01935-24. doi: 10.1128/spectrum.01935-24 (PMC11619318; doi:10.1128/spectrum.01935-24)

Supplementary material

Global prevalence of *Mycobacterium massiliense* strains with recombinant *rpoB* genes (Rec-mas) horizontally transferred from *Mycobacterium abscessus*: Two major types, dominant circulating clone 7 and MLST ST46 sequence type

Dong Hyun Kim, Hyejun Seo, Sangkwon Jung and Bum-Joon Kim *

Figure S1. Functional categories of the recombinant genes in the core genomes of DCC7 and ST46.

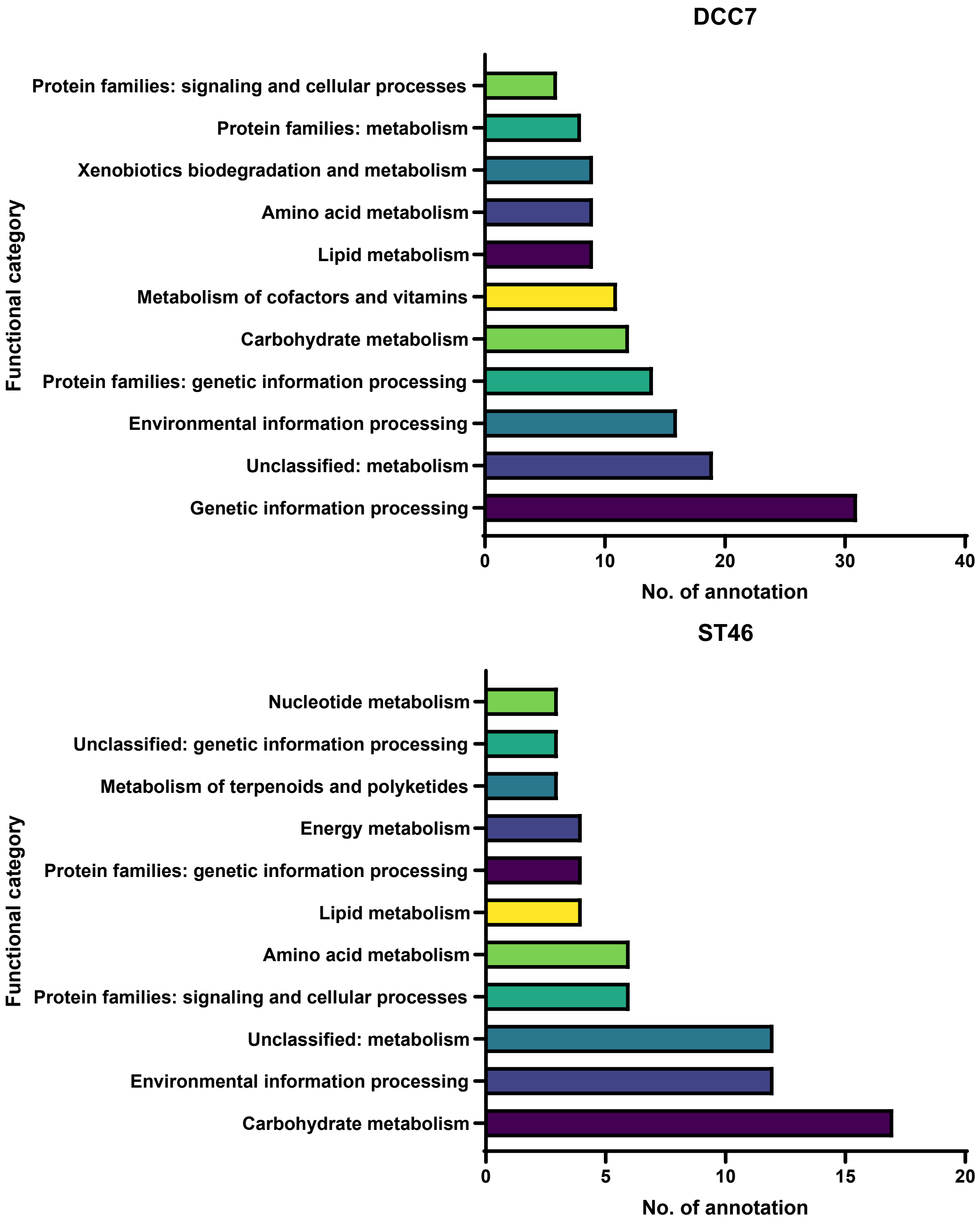

Supplement: Fig. S1 — Functional category of recombinant genes. [file spectrum.01935-24-s0001.pdf]
